# The genome sequence of the German wasp, *Vespula germanica *(Fabricius, 1793)

**DOI:** 10.12688/wellcomeopenres.17703.1

**Published:** 2022-02-15

**Authors:** Liam M. Crowley, University of Oxford and Wytham Woods Genome Acquisition Lab, Darwin Tree of Life Barcoding collective, Wellcome Sanger Institute Tree of Life programme, Wellcome Sanger Institute Scientific Operations: DNA Pipelines collective, Tree of Life Core Informatics collective, Darwin Tree of Life Consortium

**Affiliations:** 1 Department of Zoology, University of Oxford, Oxford, UK

**Keywords:** Vespula germanica, German wasp, genome sequence, chromosomal, Hymenoptera

## Abstract

We present a genome assembly from an individual female *Vespula germanica *(the German wasp; Arthropoda; Insecta; Hymenoptera; Vespidae). The genome sequence is 206 megabases in span. The majority of the assembly (98.55%) is scaffolded into 25 chromosomal pseudomolecules. The mitochondrial genome was also assembled and is 18.0 kilobases in length. Annotation of the genome assembly on Ensembl has identified 12,361 protein-coding genes.

## Species taxonomy

Eukaryota; Metazoa; Ecdysozoa; Arthropoda; Hexapoda; Insecta; Pterygota; Neoptera; Endopterygota; Hymenoptera; Apocrita; Aculeata; Vespoidea; Vespidae; Vespinae; Vespula; *Vespula germanica* (Fabricius, 1793) (NCBI:txid30212).

## Background

The German wasp, *Vespula germanica,* is a common and widespread species of social wasp throughout the palearctic. It has been introduced and become established in North America, South America, South Africa, Australia and New Zealand ( Archer, 1998), where in some instances it has become a significant invasive species ( Beggs *et al.*, 2011; Eloff *et al.*, 2020; Masciocchi & Corley, 2013). It is one of the most common and widespread social wasps in the UK, although populations have declined markedly since the 1970s ( Archer, 2001). It is found throughout a wide variety of habitats, including urban areas. This species is eusocial, living in colonies with a reproductive queen (16–19 mm), sterile workers (12–14 mm) and reproductive males (14–17 mm) ( Archer & Turner, 2014). Adults are strikingly marked yellow and black, with clear yellow genae that distinguish it from the very similar *Vespula vulgaris*. The clypeal markings varyingly consist of an incomplete dorso-ventral stripe plus two spots or three spots, unlike the anchor-like marking that is typical of *V. vulgaris*.

Colonies are annual in the UK, typically producing up to around 8000 cells ( Archer & Turner, 2014). Nests are mostly constructed underground, particularly within old rodent holes, but can also be found in enclosed aerial situations such as roof spaces, sheds and thick hedges ( Archer, 1998). Nests are constructed out of a paper-like substance produced from macerated wood fibres mixed with saliva. The nest consists of hexagonal cells arranged into eight to nine combs, covered by a nest envelope. The nest envelope of this species is grey in colour due to the well-weathered wood used to make the pulp.

Overwintered queens typically emerge from overwintering diapause around mid-March, nest founding occurs in May, and the first workers appear from early June. Males and new queens are produced from August, and mating occurs in September. Queens are typically polyandrous ( Goodisman *et al.*, 2002). Nests usually persist until around November, although some may last through the winter until early spring. There is large variation in population sizes between years, with cyclical abundance and scarcity of individuals ( Archer, 1985).

This species is generalist opportunistic predator and scavenger, with workers preying on a wide range of insect and other arthropod species and scavenging from large protein sources, such as carrion ( D’adamo & Lozada, 2007). Food is malaxated before being carried back to the nest to be fed to the developing brood. Adults feed on carbohydrate rich substances including nectar, sap, honeydew and secretions from the larvae. The propensity of adults to visit flowers, particularly shallow blooms such as ivy ( *Hedera helix*) and umbellifers, means this species may act an important pollinator. Workers have been shown to exhibit limited temporal polyethism, with nest work, pulp foraging, carbohydrate foraging, and protein foraging sequentially performed by workers as they age ( Hurd *et al.*, 2007).

## Genome sequence report

The genome was sequenced from a single female *V. germanica* collected from Wytham Woods, Oxfordshire (biological vice-county: Berkshire), UK (latitude 51.770, longitude -1.339). A total of 72-fold coverage in Pacific Biosciences single-molecule long reads and 181-fold coverage in 10X Genomics read clouds were generated. Primary assembly contigs were scaffolded with chromosome conformation Hi-C data. Manual assembly curation corrected 13 missing/misjoins and removed one haplotypic duplication, reducing the scaffold number by 21.74%, and increasing the scaffold N50 by 9.19%.

**Figure 1.**
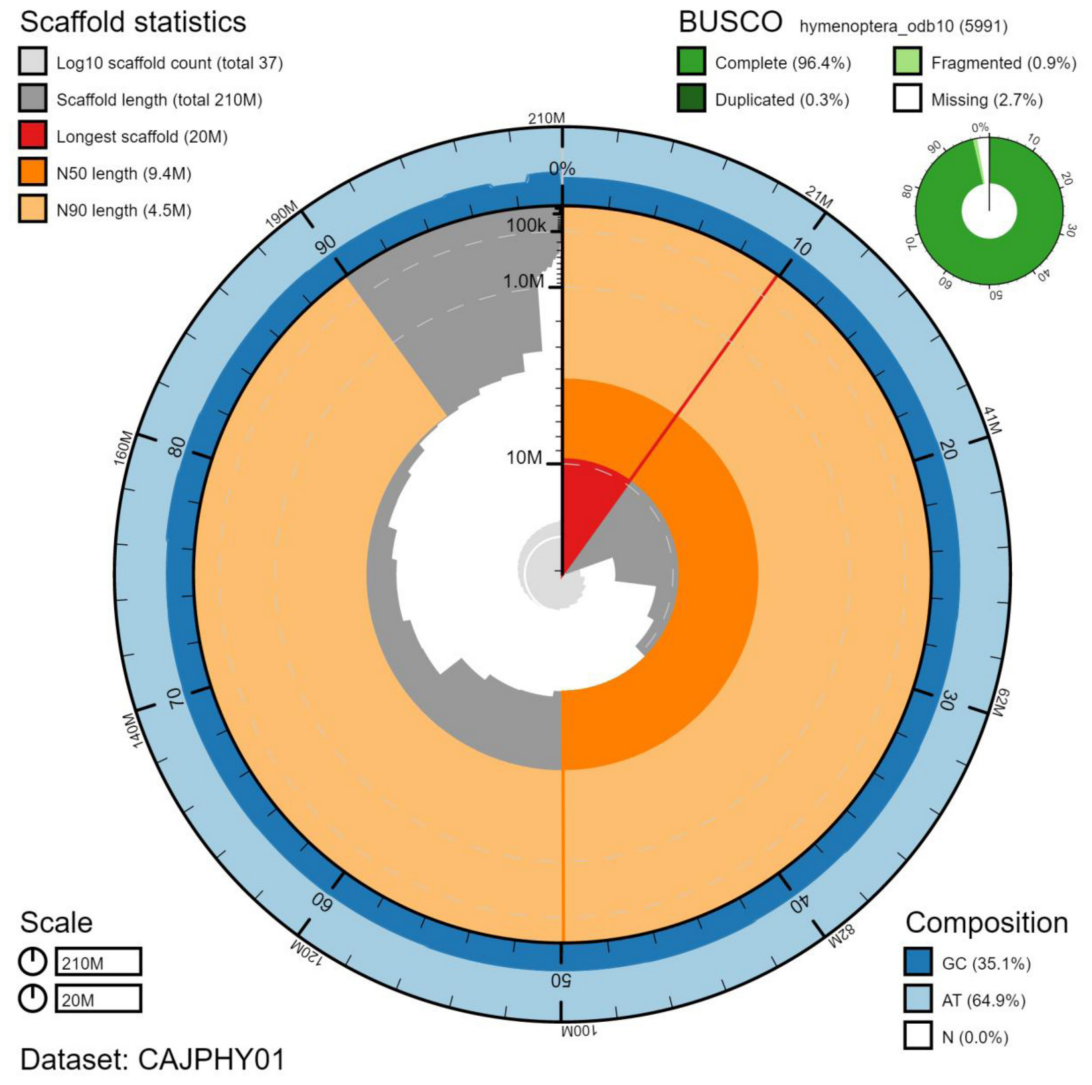
Genome assembly of *Vespula germanica*, iyVesGerm1.1: metrics. The BlobToolKit Snailplot shows N50 metrics and BUSCO gene completeness. The main plot is divided into 1,000 size-ordered bins around the circumference with each bin representing 0.1% of the 205,789,424 bp assembly. The distribution of scaffold lengths is shown in dark grey with the plot radius scaled to the longest scaffold present in the assembly (20,389,060 bp, shown in red). Orange and pale-orange arcs show the N50 and N90 scaffold lengths (9,441,317 and 4,478,551 bp), respectively. The pale grey spiral shows the cumulative scaffold count on a log scale with white scale lines showing successive orders of magnitude. The blue and pale-blue area around the outside of the plot shows the distribution of GC, AT and N percentages in the same bins as the inner plot. A summary of complete, fragmented, duplicated and missing BUSCO genes in the hymenoptera_odb10 set is shown in the top right. An interactive version of this figure is available at https://blobtoolkit.genomehubs.org/view/CAJPHY01/dataset/CAJPHY01/snail.

The final assembly has a total length of 206Mb in 36 sequence scaffolds with a scaffold N50 of 9.4 Mb ( Table 1). Of the assembly sequence, 98.55% was assigned to 25 chromosomal-level scaffolds (numbered by sequence length) ( Figure 1– Figure 4; Table 2). The assembly has a BUSCO v5.1.2 ( Manni *et al.*, 2021) completeness of 96.4% (single 96.1%, duplicated 0.3%) using the hymenoptera_odb10 reference set (n=5991). While not fully phased, the assembly deposited is of one haplotype. Contigs corresponding to the second haplotype have also been deposited.

**Table 1.** Genome data for *Vespula germanica*, iyVesGerm1.1.

*Project accession data*	
Assembly identifier	iyVesGerm1.1
Species	*Vespula germanica*
Specimen	iyVesGerm1
NCBI taxonomy ID	NCBI:txid30212
BioProject	PRJEB43741
BioSample ID	SAMEA7520501
Isolate information	Female, head/thorax
*Raw data accessions*	
PacificBiosciences SEQUEL II	ERR6635596
10X Genomics Illumina	ERR6054558-ERR6054561
Hi-C Illumina	ERR6054562-ERR6054564
PolyA RNA-Seq Illumina	ERR6286717
*Genome assembly*	
Assembly accession	GCA_905340365.1
*Accession of alternate haplotype*	GCA_905340295.1
Span (Mb)	206
Number of contigs	56
Contig N50 length (Mb)	6.6
Number of scaffolds	36
Scaffold N50 length (Mb)	9.4
Longest scaffold (Mb)	20.4
BUSCO * genome score	C:96.4%[S:96.1%,D:0.3%], F:0.9%,M:2.7%,n:5991
*Genome annotation*	
Number of protein-coding genes	12,361
Average length of coding sequence (bp)	1,553.05
Average number of exons per transcript	6.94
Average exon size (bp)	331.04
Average intron size (bp)	1,355.58

*BUSCO scores based on the hymenoptera_odb10 BUSCO set using v5.1.2. C= complete [S= single copy, D=duplicated], F=fragmented, M=missing, n=number of orthologues in comparison. A full set of BUSCO scores is available at https://blobtoolkit.genomehubs.org/view/CAJPHY01/dataset/CAJPHY01/busco.

**Figure 2.**
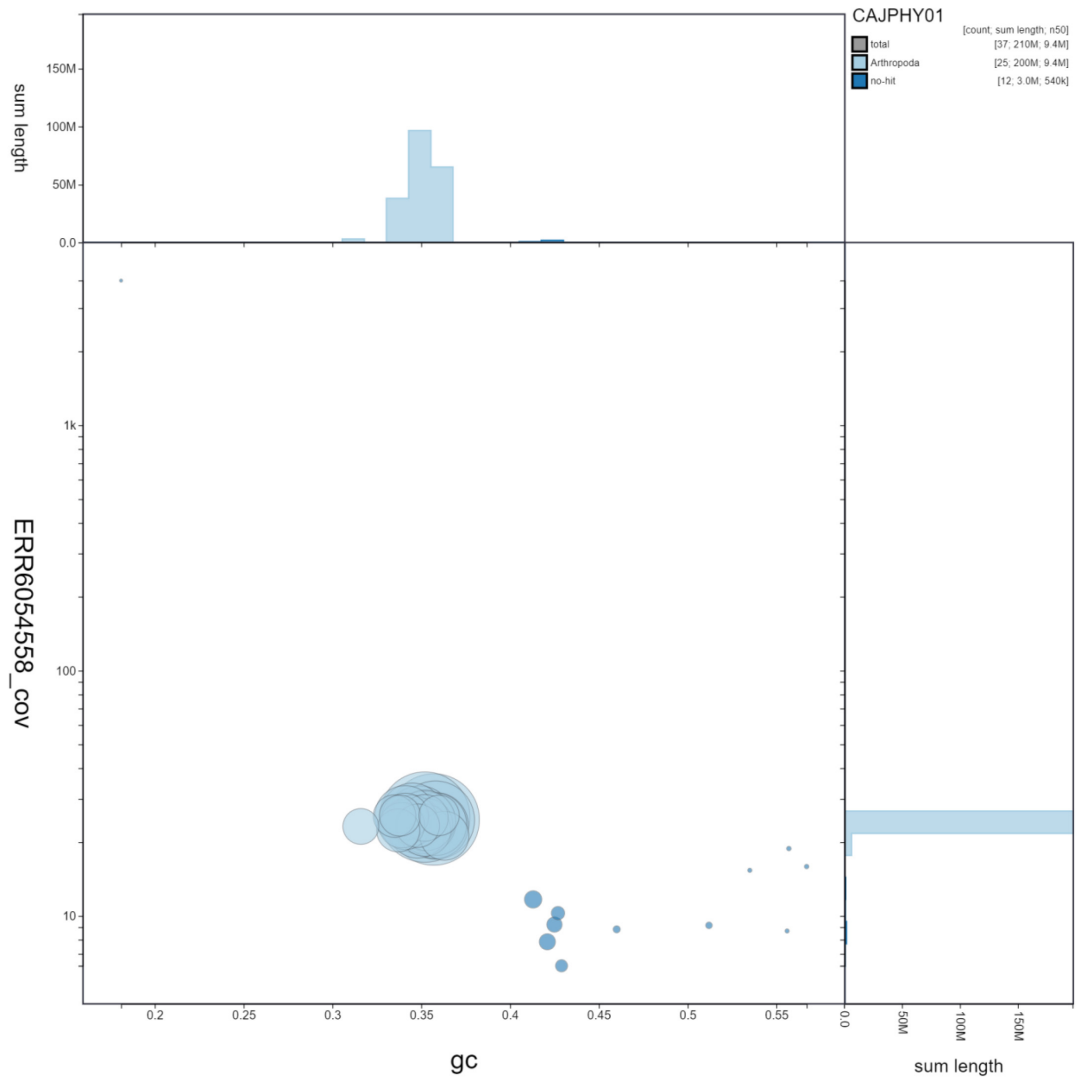
Genome assembly of *Vespula germanica*, iyVesGerm1.1: GC coverage. BlobToolKit GC-coverage plot. Scaffolds are coloured by phylum. Circles are sized in proportion to scaffold length. Histograms show the distribution of scaffold length sum along each axis. An interactive version of this figure is available at https://blobtoolkit.genomehubs.org/view/CAJPHY01/dataset/CAJPHY01/blob.

**Figure 3.**
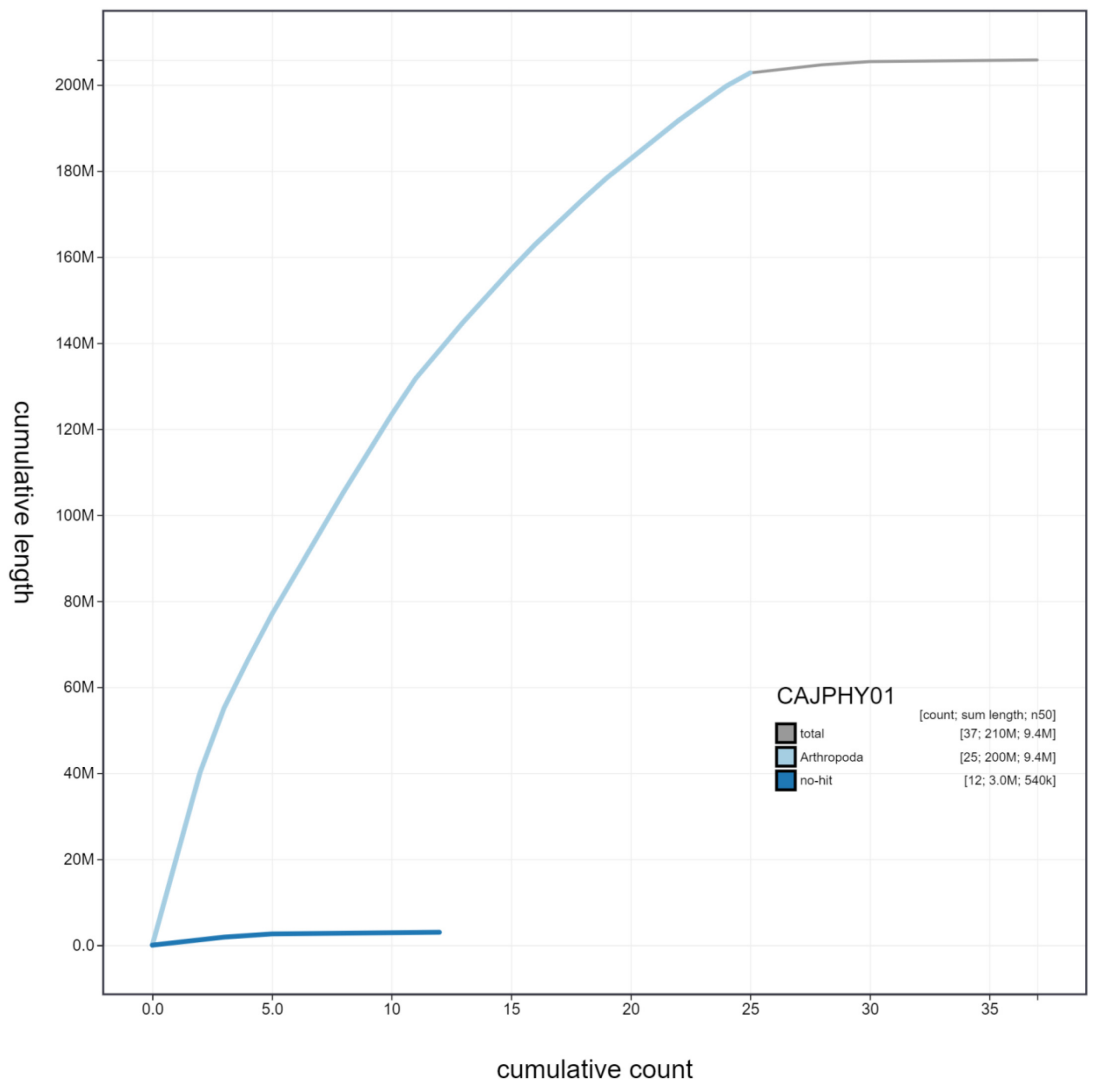
Genome assembly of *Vespula germanica*, iyVesGerm1.1: cumulative sequence. BlobToolKit cumulative sequence plot. The grey line shows cumulative length for all scaffolds. Coloured lines show cumulative lengths of scaffolds assigned to each phylum using the buscogenes taxrule. An interactive version of this figure is available at https://blobtoolkit.genomehubs.org/view/CAJPHY01/dataset/CAJPHY01/cumulative.

**Figure 4.**
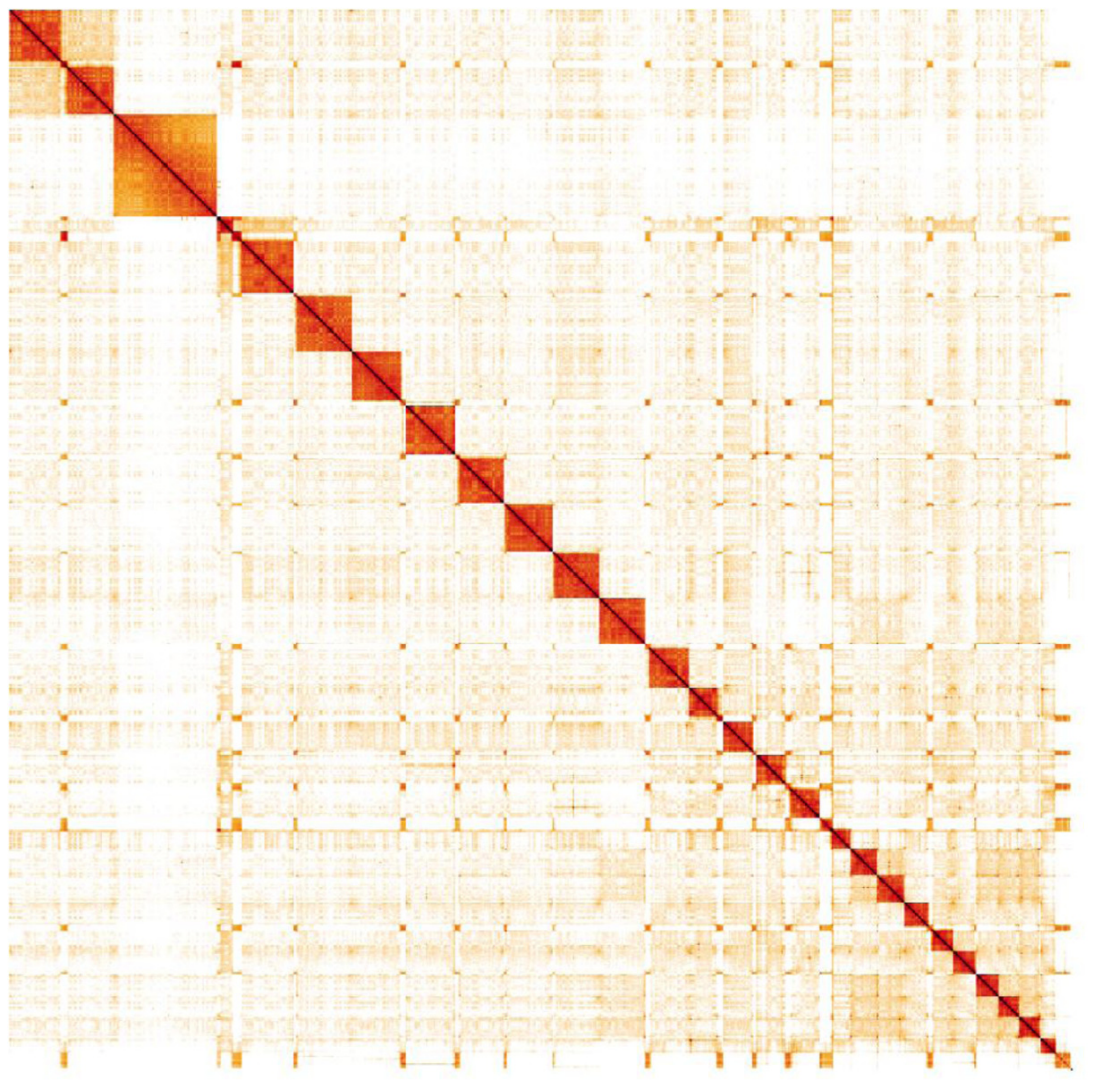
Genome assembly of *Vespula germanica*, iyVesGerm1.1: Hi-C contact map. Hi-C contact map of the iyVesCrab1.1 assembly, visualised in HiGlass. Chromosomes are shown in size order from left to right and top to bottom.

**Table 2.** Chromosomal pseudomolecules in the genome assembly of *Vespula germanica*, iyVesGerm1.1.

INSDC accession	Chromosome	Size (Mb)	GC%
HG996528.1	1	20.39	35.7
HG996529.1	2	19.91	35.2
HG996530.1	3	14.87	35.8
HG996531.1	4	11.21	34.9
HG996532.1	5	10.58	35.8
HG996533.1	6	9.48	34.5
HG996534.1	7	9.47	35.8
HG996535.1	8	9.44	35.2
HG996536.1	9	9.01	35.2
HG996537.1	10	8.94	34.1
HG996538.1	11	8.41	34.8
HG996539.1	12	6.60	35.2
HG996540.1	13	6.55	35.2
HG996541.1	14	6.12	34.5
HG996542.1	15	6.11	34.1
HG996543.1	16	5.79	36.3
HG996544.1	17	5.26	35.2
HG996545.1	18	5.24	34.1
HG996546.1	19	4.99	33.9
HG996547.1	20	4.56	34.8
HG996548.1	21	4.48	33.7
HG996549.1	22	4.34	33.5
HG996550.1	23	4.05	33.8
HG996551.1	24	3.92	36.0
HG996552.1	25	3.10	31.6
HG996553.1	MT	0.02	18.1
-	Unplaced	2.97	43.3

## Genome annotation report

The iyVesGerm1.1 genome assembly has been annotated using the Ensembl rapid annotation pipeline ( Table 1; https://rapid.ensembl.org/Vespula_germanica_GCA_905340365.1/). The resulting annotation includes 34,067 transcribed RNAs from 12,361 protein-coding and 4,967 non-coding genes. There are 2.26 coding transcripts per gene and 6.94 exons per transcript.

## Methods

### Sample acquisition and DNA extraction

A single female *V. germanica* (iyVesGerm1) was collected from Wytham Woods, Oxfordshire (biological vice-county: Berkshire), UK (latitude 51.770, longitude -1.339) by Liam Crowley, University of Oxford, using a net. The sample was identified by the same individual and snap-frozen on dry ice. Unfortunately, no image of the sequenced specimen is available.

DNA was extracted from head/thorax tissue of iyVeGerm1 at the Wellcome Sanger Institute (WSI) Scientific Operations core from the whole organism using the Qiagen MagAttract HMW DNA kit, according to the manufacturer’s instructions. RNA was extracted from remaining head/thorax tissue of iyVesGerm1 in the Tree of Life Laboratory at the WSI using TRIzol (Invitrogen), according to the manufacturer’s instructions. RNA was then eluted in 50 μl RNAse-free water and its concentration assessed using a Nanodrop spectrophotometer and Qubit Fluorometer using the Qubit RNA Broad-Range (BR) Assay kit. Analysis of the integrity of the RNA was done using Agilent RNA 6000 Pico Kit and Eukaryotic Total RNA assay.

### Sequencing

Pacific Biosciences HiFi circular consensus and 10X Genomics Chromium read cloud sequencing libraries were constructed according to the manufacturers’ instructions. Poly(A) RNA-Seq libraries were constructed using the NEB Ultra II RNA Library Prep kit. Sequencing was performed by the Scientific Operations core at the Wellcome Sanger Institute on Pacific Biosciences SEQUEL II (HiFi), Illumina HiSeq X (10X) and Illumina HiSeq 4000 (RNA-Seq) instruments. Hi-C data were generated from head/thorax tissue of iyVesGerm1 using the Arima v2 kit and sequenced on HiSeq X.

### Genome assembly

Assembly was carried out with Hifiasm ( Cheng *et al.*, 2021). Haplotypic duplication was identified and removed with purge_dups ( Guan *et al.*, 2020). One round of polishing was performed by aligning 10X Genomics read data to the assembly with longranger align, calling variants with freebayes ( Garrison & Marth, 2012). The assembly was then scaffolded with Hi-C data ( Rao *et al.*, 2014) using SALSA2 ( Ghurye *et al.*, 2019). The assembly was checked for contamination and corrected using the gEVAL system ( Chow *et al.*, 2016) as described previously ( Howe *et al.*, 2021). Manual curation was performed using gEVAL, HiGlass ( Kerpedjiev *et al.*, 2018) and Pretext. The mitochondrial genome was assembled using MitoHiFi ( Uliano-Silva *et al.*, 2021), which performed annotation using MitoFinder ( Allio *et al.*, 2020). The genome was analysed and BUSCO scores generated within the BlobToolKit environment ( Challis *et al.*, 2020). Table 3 contains a list of all software tool versions used, where appropriate.

**Table 3.** Software tools used.

Software tool	Version	Source
Hifiasm	0.12	Cheng *et al.*, 2021
purge_dups	1.2.3	Guan *et al.*, 2020
SALSA2	2.2	Ghurye *et al.*, 2019
longranger align	2.2.2	https://support.10xgenomics.com/genome-exome/ software/pipelines/latest/advanced/other-pipelines
freebayes	v1.3.1-17-gaa2ace8	Garrison & Marth, 2012
MitoHiFi	1.0	https://github.com/marcelauliano/MitoHiFi
gEVAL	N/A	Chow *et al.*, 2016
HiGlass	1.11.6	Kerpedjiev *et al.*, 2018
PretextView	0.1.x	https://github.com/wtsi-hpag/PretextView
BlobToolKit	2.6.4	Challis *et al.*, 2020

### Ethics/compliance issues

The materials that have contributed to this genome note have been supplied by a Darwin Tree of Life Partner. The submission of materials by a Darwin Tree of Life Partner is subject to the Darwin Tree of Life Project Sampling Code of Practice. By agreeing with and signing up to the Sampling Code of Practice, the Darwin Tree of Life Partner agrees they will meet the legal and ethical requirements and standards set out within this document in respect of all samples acquired for, and supplied to, the Darwin Tree of Life Project. Each transfer of samples is further undertaken according to a Research Collaboration Agreement or Material Transfer Agreement entered into by the Darwin Tree of Life Partner, Genome Research Limited (operating as the Wellcome Sanger Institute), and in some circumstances other Darwin Tree of Life collaborators.

## Data availability

European Nucleotide Archive: Vespula germanica (German wasp). Accession number PRJEB43741; https://identifiers.org/ena.embl/PRJEB43741.

The genome sequence is released openly for reuse. The *V. germanica* genome sequencing initiative is part of the Darwin Tree of Life (DToL) project. All raw sequence data and the assembly have been deposited in INSDC databases. Raw data and assembly accession identifiers are reported in Table 1.

## Author information

Members of the University of Oxford and Wytham Woods Genome Acquisition Lab are listed here: https://doi.org/10.5281/zenodo.5746938.

Members of the Darwin Tree of Life Barcoding collective are listed here: https://doi.org/10.5281/zenodo.5744972.

Members of the Wellcome Sanger Institute Tree of Life programme are listed here: https://doi.org/10.5281/zenodo.5744840.

Members of Wellcome Sanger Institute Scientific Operations: DNA Pipelines collective are listed here: https://doi.org/10.5281/zenodo.5746904.

Members of the Tree of Life Core Informatics collective are listed here: https://doi.org/10.5281/zenodo.5743293.

Members of the Darwin Tree of Life Consortium are listed here: https://doi.org/10.5281/zenodo.5638618.
