# Supplementary material for: The genome sequence of the German wasp, Vespula germanica (Fabricius, 1793)
Source: Wellcome Open Res. 2022 Feb 15;7:60. [Version 1] doi: 10.12688/wellcomeopenres.17703.1 (PMC9471221; doi:10.12688/wellcomeopenres.17703.1)
Supplement: Peer review history

# Peer review: Reviewer response for version 1

**DOI:** 10.21956/wellcomeopenres.19590.r51963

**Published:** 2022-09-13

**Authors:** Rodolpho S. T. Menezes

**Affiliations:** 1 Universidade Federal de Santa Maria, Santa Maria, RS, Brazil

This short genome report describes the genome sequencing of the German wasp, *Vespula germanica*. The work begins with a background about the natural history as well as some morphological features of this social wasp are commented. The DNA extraction, sequencing, and assembly process are well described. The genome assembly reported will be interesting for further studies.

- I think in the sections “Genome sequence report” and “Genome annotation report”, information about the mitochondrial genome should be better described.
- Keywords: Please be in alphabetic order. Change "chromosomal" to "chromosome".

Are sufficient details of methods and materials provided to allow replication by others?

Yes

Is the rationale for creating the dataset(s) clearly described?

Yes

Are the datasets clearly presented in a useable and accessible format?

Partly

Are the protocols appropriate and is the work technically sound?

Yes

Reviewer Expertise:

Phylogenomics, Phylogeography, and Cytogenetics.

I confirm that I have read this submission and believe that I have an appropriate level of expertise to confirm that it is of an acceptable scientific standard.

# Peer review: Reviewer response for version 1

**DOI:** 10.21956/wellcomeopenres.19590.r48729

**Published:** 2022-03-16

**Authors:** Volker P. Brendel

**Affiliations:** 1 Department of Biology, Indiana University, Bloomington, IN, USA

The Data Note provides a succinct review of the genome assembly and annotation efforts by the author group concerning the German wasp. The approaches taken are standard practice and well enough documented to serve as a template for similar genome projects. All the data (raw sequence reads and derived pseudomolecules and hypothetical annotation products) are easily accessible in public databases. The genome assembly and annotation provides a starting point for further phylogenetic and functional studies not pursued in this paper - and rightly so, as such studies should encompass multiple similar data sets.

Are sufficient details of methods and materials provided to allow replication by others?

Yes

Is the rationale for creating the dataset(s) clearly described?

Yes

Are the datasets clearly presented in a useable and accessible format?

Yes

Are the protocols appropriate and is the work technically sound?

Yes

Reviewer Expertise:

computational genomics

I confirm that I have read this submission and believe that I have an appropriate level of expertise to confirm that it is of an acceptable scientific standard.
